# Hepatic Epigenetic Reprogramming After Liver Resection in Offspring Alleviates the Effects of Maternal Obesity

**DOI:** 10.3389/fcell.2022.830009

**Published:** 2022-03-31

**Authors:** Lais A. de Paula Simino, Marina Figueiredo Fontana, Thais de Fante, Carolina Panzarin, Letícia Martins Ignacio-Souza, Marciane Milanski, Marcio Alberto Torsoni, Mina Desai, Michael G. Ross, Adriana Souza Torsoni

**Affiliations:** ^1^ Laboratory of Metabolic Disorders, School of Applied Sciences, University of Campinas—UNICAMP, Limeira, Brazil; ^2^ The Lundquist Institute and David Geffen School of Medicine at Harbor-UCLA Medical Center, University of California, Los Angeles, Los Angeles, CA, United States

**Keywords:** partial hepatectomy, microRNAs, metabolic programming, DOHaD, obesity, liver regeneration, NAFLD

## Abstract

Obesity has become a public health problem in recent decades, and during pregnancy, it can lead to an increased risk of gestational complications and permanent changes in the offspring resulting from a process known as metabolic programming. The offspring of obese dams are at increased risk of developing non-alcoholic fatty liver disease (NAFLD), even in the absence of high-fat diet consumption. NAFLD is a chronic fatty liver disease that can progress to extremely severe conditions that require surgical intervention with the removal of the injured tissue. Liver regeneration is necessary to preserve organ function. A range of pathways is activated in the liver regeneration process, including the Hippo, TGFβ, and AMPK signaling pathways that are under epigenetic control. We investigated whether microRNA modulation in the liver of the offspring of obese dams would impact gene expression of Hippo, TGFβ, and AMPK pathways and tissue regeneration after partial hepatectomy (PHx). Female Swiss mice fed a standard chow or a high-fat diet (HFD) before and during pregnancy and lactation were mated with male control mice. The offspring from control (CT-O) and obese (HF-O) dams weaned to standard chow diet until day 56 were submitted to PHx surgery. Prior to the surgery, HF-O presented alterations in miR-122, miR-370, and Let-7a expression in the liver compared to CT-O, as previously shown, as well as in its target genes involved in liver regeneration. However, after the PHx (4 h or 48 h post-surgery), differences in gene expression between CT-O and HF-O were suppressed, as well as in microRNA expression in the liver. Furthermore, both CT-O and HF-O presented a similar regenerative capacity of the liver within 48 h after PHx. Our results suggest that survival and regenerative mechanisms induced by the partial hepatectomy may overcome the epigenetic changes in the liver of offspring programmed by maternal obesity.

## Introduction

Obesity is a global epidemic as its prevalence has more than tripled since 1975 (World Health Organization, 2018). Excessive fat accumulation is considered a major risk factor for several chronic diseases, such as non-alcoholic fatty liver disease (NAFLD) (Younossi et al., 2016). NAFLD is defined as an ectopic lipid accumulation within the hepatocytes up to 5% in the absence of drugs or alcohol consumption (Lindenmeyer and McCullough, 2018), which can progress to stages with associated inflammation and fibrosis, fatally causing liver failure (Koppe, 2014; Bellentani and Med Stefano Bellentani, 2017). Recent studies have demonstrated that NAFLD development can be triggered during fetal and early stages of life, as a consequence of exposure to a maternal obesogenic environment, such as a high-fat diet (HFD) consumption (Hoover et al., 1987; Fernandez-Twinn et al., 2015; Simino et al., 2021). In addition, the HFD consumption by dams predisposes offspring to higher body weight and adiposity, dyslipidemia, and insulin resistance at different stages of life (Melo et al., 2014; Fante et al., 2016; Lemes et al., 2018; Castro-Rodríguez et al., 2021). The development of these metabolic abnormalities in the offspring, as well as NAFLD, has shown to be under epigenetic control (Benatti et al., 2014; Fernandez-Twinn et al., 2015; Bianco-Miotto et al., 2017; Simino et al., 2021).

Recently, microRNA (miRNA) modulation has been associated with offspring outcomes programmed by maternal obesity during gestation and/or lactation (Benatti et al., 2014; Simino et al., 2017; Sun et al., 2020; Simino et al., 2021). miRNAs are small molecules of non-coding RNAs that mostly act as a target mRNA silencer (Correia de Sousa et al., 2019). Weanling and adult offspring of obese dams have altered hepatic miRNAs that impact the key lipid enzymes causing fat liver accumulation (Benatti et al., 2014; Simino et al., 2017). We have shown that mice offspring programmed by maternal obesity exhibit higher levels of miR-370 and Let-7 that target *Cpt1a* and *Prkaa2* (AMPKα2 encoding gene), respectively, possibly resulting in lower fat oxidation in the liver (Simino et al., 2017; Simino et al., 2021). In addition, the offspring of maternal obesity have reduced levels of hepatic miR-122, which is the main miRNA in the liver that targets key genes involved in triglyceride synthesis, such as *Agpat*, thus promoting lipogenesis (Simino et al., 2017). As a result of this, the offspring of obese dams display an ectopic fat liver accumulation and become predisposed to an increased risk of metabolic syndrome development.

The progression of NAFLD to more severe conditions may require surgical intervention, such as bariatric surgery, liver transplantation, or partial hepatectomy (PHx), the latter involving the removal of the injured tissue to preserve the organ functions (Koppe, 2014; Shingina et al., 2019; Lassailly et al., 2020). The experimental studies have shown that obese mice with NAFLD have a major incidence of complications and death after liver resection surgery or liver transplantation (Todo et al., 1989; Behrns et al., 1998; Murata et al., 2007; Jin et al., 2019; Kumar et al., 2019). Human epidemiological evidence has demonstrated that obese patients have lower liver regeneration index and kinetic growth rate of the liver after major hepatic resection than eutrophic patients (Truant et al., 2013; Amini et al., 2016).

Liver resection through PHx promotes great cellular stress, inducing inflammatory cytokine production and release which signal the initial phase of the liver regeneration with the G1 cell cycle, and subsequent activation of several genes involved in cell replication (Li et al., 2001; Mao et al., 2014). We have recently shown that mice offspring programmed by maternal obesity during gestation and lactation have delayed regenerative response after PHx since they present lower IL-6 levels, Ki67 labeling, cells in S-phase, and Cyclin D1 content as compared to the offspring of control dams. However, even when rechallenged to HFD in adult life, PHx had lower impact on the survival rate of the offspring of obese dams, since they reach successful liver regeneration as the offspring of control dams (Fante et al., 2021).

Although liver regeneration processes have been largely studied, little is known about the influence of hepatic miRNA modulation on liver regeneration after partial hepatectomy in offspring programmed by maternal obesity. We investigated whether the hepatic miRNA alterations previously reported in offspring programmed by maternal obesity would impact key gene expression that could affect the tissue regeneration processes after PHx.

## Methods

### Experimental Design

Female and male Swiss mice (5 weeks old, *n* = 10 females per group) were obtained from the Animal Breeding Center at the University of Campinas (CEMIB/Unicamp, Brazil). The experiments were approved by the Committee for Ethics in Animal Experimentation (protocol #4594-1/2017) at the State University of Campinas–UNICAMP and were performed in accordance with the guidelines of the Brazilian College for Animal Experimentation (COBEA) and with the National Institutes of Health guide for the care and use of laboratory animals (NIH Publications #8023, revised 1978). The females were randomly separated into two groups: the standard chow-fed group (CT, NUVILAB^®^ Cr-1; Nuvital; 3.5 kcal/g, 9.5% of energy from fat) and a high-fat diet-fed group (HF for growth; AIN-93G; 4.6 kcal/g, 45% of energy from fat) both *ad libitum* during the adaptation period (4 weeks before mating), gestation, and lactation. The HF diet was prepared according to previous studies (Simino et al., 2017), and only obesity-prone females were included in the HF group (80% belonging to the cohort described by Fante et al., 2021). All mice had free access to water and were maintained in individual polypropylene micro-isolators at 22 ± 1°C with lights on from 06:00 to 18:00 h. Swiss males from the same age fed CT diet were used for mating (2 females:1 male). After birth, both litters of control dams (CT-O) and HF dams (HF-O) were adjusted to eight male pups per dam, and females were discarded. When the number of males was not enough, females were kept until weaning. After weaning, the offspring were fed CT diet *ad libitum* until 8 weeks of age (p56), when they were submitted to a PHx procedure. Euthanasia was undertaken in all mice after intraperitoneal anesthesia (ketamine: xylazine: diazepam ratio 3:2:2) followed decapitation. One male pup of each litter was used for analysis.

### Partial Hepatectomy

After inhalation anesthesia with isoflurane and oxygen (2%:2L/min), a PHx procedure was performed at p56 to remove ⅔ of the liver and assess the hepatic regenerative capacity of offspring (Mitchell and Willenbring, 2008). After surgery, the animals received analgesia every 24 h until the sacrifice (carprofen- 10 mg/kg of body weight). The left lobe was removed during surgery and used for time 0 h analyses (baseline), and the right lobe was used for analyses at 4 and 48 h after PHx. To avoid discrepancies caused by methodological artifacts, all the surgeries were conducted by the same researcher and the liver samples with hemorrhage resulting from stenosis of the suprahepatic vena cava were excluded, as stated by Mitchell and Willenbring (2008) *.*

### Body Composition and Serum Biochemical Analysis

The following analyses were performed in dams and offspring from both groups. Bodyweight was measured weekly, and adiposity was evaluated by the ratio of epididymal white adipose tissue mass, collected after the euthanasia, and total body weight. Fasting glucose was assessed using the Accu-Chek Performa glucometer (Roche^®^, Switzerland; detection limit: 10 mg/dl). Triglycerides (TG) and cholesterol (CHOL) serum content were quantified using enzymatic kits (Laborlab, Brazil; detection limit: 0.8–3.0 mg/dl), according to the manufacturer’s guidelines. Alanine aminotransferase (ALT) and aspartate aminotransferase (AST) were analyzed using a kinetic spectrophotometric method in offspring serum in the baseline and after 4 and 48 h of PHx according to the manufacturer’s guidelines (ALT: Labtest ref.: 108; AST: Labtest ref.: 109, Brazil; detection limit: 1.75U/L). Serum insulin was determined using the Rat/Mouse Insulin ELISA Kit (Millipore, Germany; 1.3–4.0 ng/ml sensitivity).

### *In Silico* Analysis of miRNA Potential Targets

*In silico* analyses were performed to identify predicted targets genes involved in cell cycle, growth, and proliferation in *Mus musculus* considering those that match with miRNAs previously described as altered in the offspring of obese dams (miR-370, Let-7, and miR-122) (Simino et al., 2017; Simino et al., 2021). The miRNA/mRNA target prediction was performed using the MiRWalk 2.0 platform* accessing a total of 12 algorithms. Interactions were considered valid when predicted by TargetScan algorithm and, at least, five other algorithms. Then, the targets were included in the DAVID platform** to select target genes involved in cell cycle and proliferation.

### Real-Time Polymerase Chain Reaction After Reverse Transcription (RT-qPCR)

The total RNA and miRNA were extracted from the liver or cells using RNAzol reagent (Sigma Aldrich) according to the manufacturer’s recommendations and quantified using NanoDrop ND-2000. Reverse transcription was performed with 3 μg of total RNA or miRNA using the High-Capacity cDNA Reverse Transcription Kit (Thermo Fisher Scientific). The relative expression of mRNAs (Smad3 ID Mm01170760_m1, Nf2 ID Mm00477771_m1, Yap1 Mm01143263_m1, Il6 ID Mm00446190_m1, Il1b ID Mm00434228_m1, Tnf ID Mm00443258_m1, Prkaa2 ID Mm01264789_m1, and Actb ID 4352341E) and miRNAs (miR-122 ID 002245, miR-370 ID 002275, Let-7a ID 000377, and U6srRNA ID 001973) was determined using a Taqman system. qPCR was performed with 20 ng of complementary DNA on the ABI 7500 Fast System. Data were expressed as relative values determined by the comparative threshold cycle method (2-ΔΔCt), according to the manufacturer’s recommendation.

### Immunoblotting

The liver samples were homogenized in RIPA buffer. 50 ug of proteins, determined using Biuret Reagent, were separated by SDS-PAGE and electrotransferred onto a nitrocellulose membrane. After blocking, the membrane was incubated overnight with antibodies to anti-SMAD2/3 (Cell Signaling Technology, mAb #9523, 1:1000), p-SMAD2/3 (Cell Signaling Technology, mAb #9520, 1:1000), YAP/TAZ (Cell Signaling Technology, mAb #8418, 1:1000), anti-AMPK (Cell Signaling Technology, #2793, 1:1000), pAMPK (Cell Signaling Technology, mAb #2535, 1:1000), and ACTIN β as endogenous control (Abcam, ab8227, 1:1000) and then incubated with a secondary antibody. Band intensity was detected by chemiluminescence in GeneGnome equipment (Syngene) and evaluated by densitometry using Scion Image software (Scion Corporation).

### Immunofluorescence

Cell proliferation was determined by ki67 immunofluorescence analysis. In brief, the liver frozen sections (7 μm) were fixed in 4% paraformaldehyde solution for 30 min, washed in PBS, and then, incubated in PBS solution containing 0.5% Triton X-100 and 0.05% SDS for 30 min. After washing, the slides were blocked with 1% albumin diluted in PBT (0.1M PBS +0.25% Triton X-100) for 2 h at room temperature. The slides were incubated with anti-KI67 (Abcam, ab15580, 1:100) primary antibody diluted in the blocking solution overnight at 4°C and secondary antibodies (Donkey anti-mouse FITC conjugated (1:250 dilution), Abcam, Cambridge, MA, United States). The fluorescence was visualized using the IMMU-Mount immunofluorescence medium (Thermo Fisher Scientific) with DAPI solution (Sigma, D9542, 1:2000) in a fluorescence optical microscope (LEICA DMI 4000B) and quantified with ImageJ software.

### Cell Culture and Cycle Assay

Cell cycle analysis was performed using the AML12 mouse hepatocyte cell line (ATCC CRL-2254). The cells were maintained according to the work of Simino et al. (2021). Briefly, the cell were cultivated in the DMEM:HAM-F12 medium (1:1, 3.15 g/L glucose), with 10% FBS, 100 U/mL penicillin, 0.1 mg/ml streptomycin, 0.005 mg/ml insulin, 0.005 mg/ml transferrin, 5 ng/ml de selenium, and 40 ng/ml dexamethasone, and incubated at 37°C in 5% CO_2_. The experiments were performed between passages 10 and 15. To determine the distribution of cell cycle stages, the cells were transfected with Let-7a mimic, inhibitor or scramble control sequence (10 nM, Ambion), or with miR-370 mimic, inhibitor, or scramble control sequence (10 nM, Ambion), along with Lipofectamine RNAimax (Invitrogen), in a serum-free culture medium, for 24 h. The transfected hepatocytes were extracted and isolated according to the work of Severgnini et al. (2012). Cells (5 × 10^5^ cells/mL) were resuspended in 20 μg/ml propidium iodide (ImmunoChemistry Technologies) diluted in 100 ul of PBS. Flow cytometric analysis was performed in two independent biological replicates from a sample pooling of two technical replicates, using a BD Accuri C6 cytometer on channels FL2 (585/40 nm) and FL3 (610/20 nm), with a total of 10,000 events counted in gate P2.

### Statistical Analysis

The results are expressed as mean ± SEM. The Shapiro–Wilk test was used to confirm normality. Student’s *t*-test was used to compare two groups, and Grubb’s test was used to determine outlier. A significance level of *p* ≤ 0.05 was established to assume differences between groups. The statistical analysis of each result is described in each figure legend. Data were analyzed using GraphPad Prism, version 8 (GraphPad Software Inc., United States).

## Results

### Offspring Programmed by Maternal Obesity Have Slight Upregulation in Inflammatory Markers After Partial Hepatectomy

At mating, HF dams presented an obese phenotype, since they had higher body weight and adiposity, accompanied by elevated serum glucose, insulin, cholesterol (CHOL), and triacylglycerol (TAG) levels (Supplementary Table S1). Similarly, the offspring of obese dams (HF-O) had a dysmetabolic phenotype before the PHx procedure, as seen by higher body weight, and adiposity, as well as increased fasting glucose, serum insulin, and TAG levels compared to the offspring of control dams (CT-O) (Supplementary Table S2).

**FIGURE 1:**
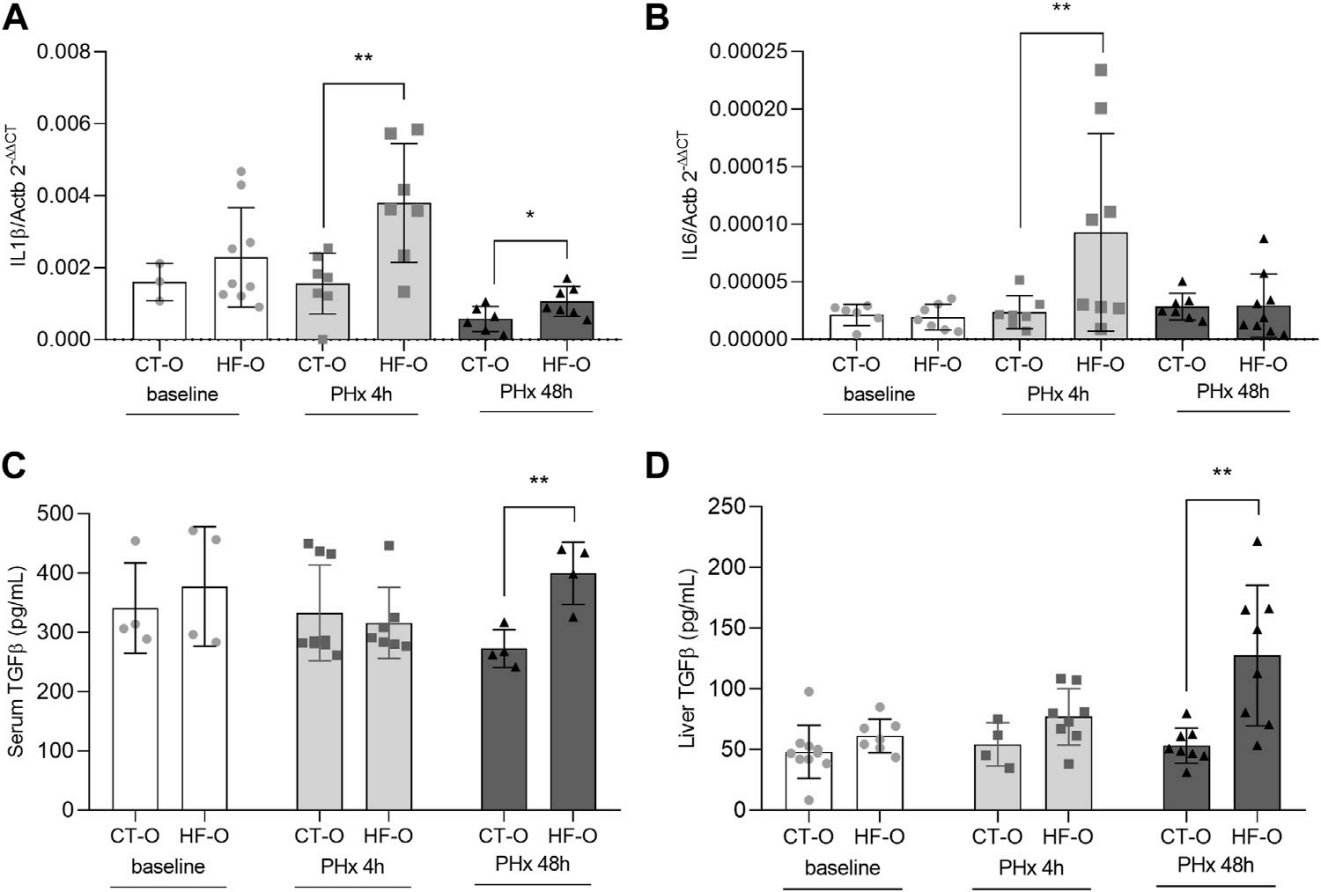
Inflammatorymarkers of the offspring of control and obese dams after partial hepatectomy. Hepatic IL1β **(A)** and IL6 **(B)** gene expression (qPCR), serum TGFβ **(C)** and liver TGFβ **(D)** protein content (ELISA) from the offspring of control (CT-O) and obese (HF-O) dams, before (baseline) or after 4 (PHx 4 h) or 48 h (PHx 48 h) of partial hepatectomy surgery. Data are plotted as individual values (dot plots), means and SEM, represented by vertical bars. n = 3–9/group. **p ≤ 0.005 after Student’s t-test (CT-O versus HF-O).

We further evaluated the inflammatory markers in the offspring after 4 and 48 h of PHx. HF-O had higher hepatic Il1b gene expression after 4 and 48 h PHx (Figure 1A) and upregulation in Il6 after 4 h PHx (Figure 1B). TGFβ was upregulated in both serum and the liver of HF-O 48 h following PHx (Figures 1C,D). Serum ALT and AST levels did not differ between groups for all time points studied, equally reflecting only the damage caused by the surgery (Supplementary Table S3).

### Key Hepatic microRNAs and Their Predicted Targets Are Altered in the Liver of Offspring Programmed by Maternal Obesity After Partial Hepatectomy

We have previously reported that offspring programmed by maternal obesity during gestation and/or lactation have modulation in key hepatic microRNAs that leads to dysregulation in energy and lipid homeostasis, such as upregulation in miR-370 and Let-7a and downregulation of miR-122 (Benatti et al., 2014; Simino et al., 2017; Simino et al., 2021). In view of this, we performed an *in silico* analysis of predicted targets of miR-370, Let-7a, and miR-122 that might play an important role in liver regeneration (Supplementary Table S4).

**FIGURE 2:**
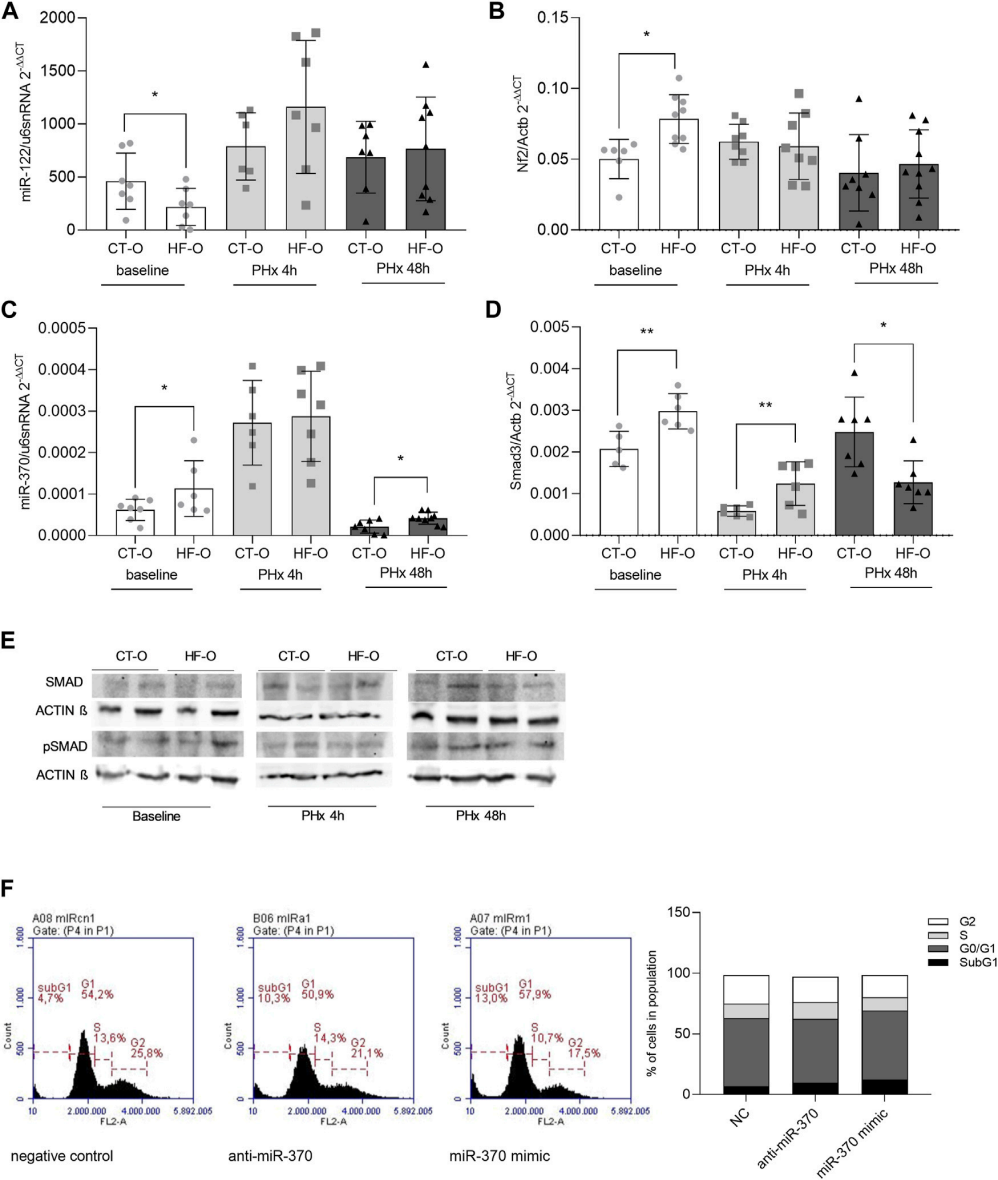
Hepatic miR-122 and miR-370 pathways in the liver of the offspring of control and obese dams after partial hepatectomy. Hepatic miR-122-5p **(A)**, Nf2 **(B)**, miR-370-3p **(C)**, and Smad3 **(D)** gene expression (qPCR) and SMAD3 and pSMAD3 immunoblotting **(E)** from the offspring of control (CT-O) and obese (HF-O) dams, before (baseline) or 4 h (PHx 4 h) or 48 h (PHx 48 h) after partial hepatectomy surgery. Data are plotted as individual values (dot plots), means and SEM, represented by vertical bars. *n* = 5–10/group. *p ≤ 0.05 and **p ≤ 0.005 after Student’s *t*-test (CT-O versus HF-O). Representative image and histogram of cell cycle distribution (*n* = 2) by flow cytometry in AML12 cells transfected with negative control, anti-miR-370, or miR-370 mimic **(F)**.

miR-122-5p is predicted to bind to *Nf2* mRNA 3′UTR (Supplementary Table S3). The *Nf2* gene codifies a protein that initiates a signaling pathway that leads to phosphorylation of the YAP/TAZ enzymatic complex that, in turn, exits the nucleus and prevents the transcription of proliferation-related genes (Nguyen-Lefebvre et al., 2021). As expected, baseline liver miR-122 levels were downregulated in HF-O offspring, whereas *Nf2* levels were upregulated. However, after 4 h or 48 h of PHx, miR-122 and *Nf2* levels in HF-O were comparable to CT-O (Figures 2A,B).

Mir-370-3p is predicted to pair with the targeting region of mRNAs 3′UTR from Smad3 and *Tgfβr2* (Supplementary Table S3). SMAD3 protein is downstream of the TGF-β receptor in the signaling pathway and acts by activating transcription factors of genes related to cellular proliferation (Nguyen-Lefebvre et al., 2021). Hepatic miR-370 was upregulated at baseline and 48 h after PHx in HF-O (Figure 2C). However, Smad3 mRNA showed a transient expression, since it was upregulated at baseline and 4 h after PHx, but downregulated 48 h after PHx in the liver of HF-O in comparison to CT-O (Figure 2D). While total-SMAD3 protein levels tend to be downregulated in the liver of HF-O 48 h after PHx, the levels of pSMAD3 did not differ between HF-O and CT-O (Figure 2E). Moreover, the transfection of AML12 cells with anti-miR or mimic for miR-370 did not lead to significant alterations in cell cycle phases, although the presence of mimic for miR-370 reduced slightly the number of cells in the S-phase (11% miR-370 mimic *vs*. 12% NC) (Figure 2F).

**FIGURE 3:**
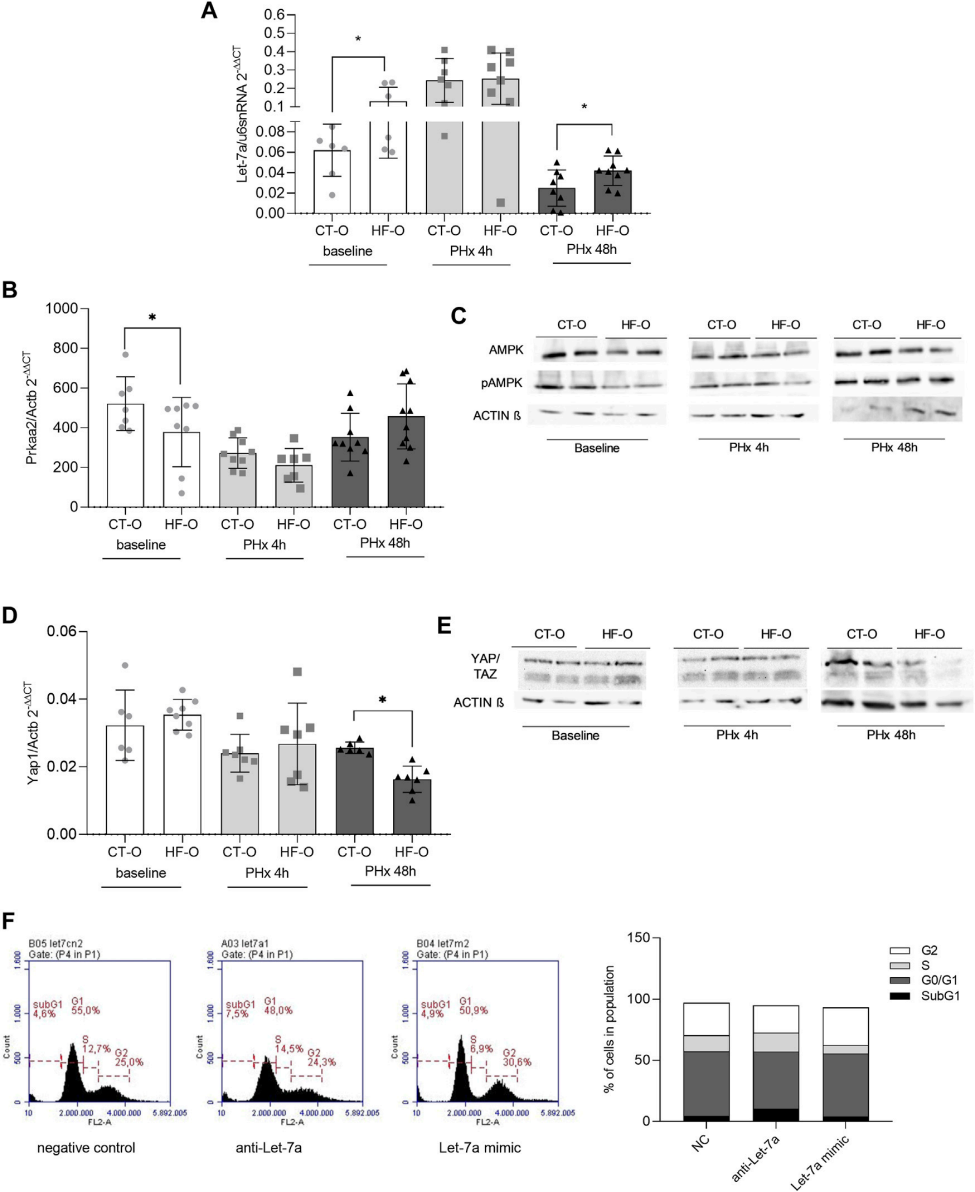
Hepatic Let-7a pathways in the liver of the offspring of control and obese dams after partial hepatectomy. Hepatic Let-7a-5p **(A)** and Prkaa2 **(B)** gene expression (qPCR), AMPKa2 and pAMPKa2 immunoblotting **(C)**, YAP1 **(D)** gene expression (qPCR), and YAP/TAZ immunoblotting **(E)** from the offspring of control (CT-O) and obese (HF-O) dams, before (baseline) or 4 h (PHx 4 h) or 48 h (PHx 48 h) after partial hepatectomy surgery. Data are plotted as individual values (dot plots), means and SEM, represented by vertical bars. n = 5–10/group. *p ≤ 0.05 and **p ≤ 0.005 after Student’s *t*-test (CT-O versus HF-O). Representative image and histogram of cell cycle distribution (*n* = 2) by flow cytometry in AML12 cells transfected with negative control, anti-Let-7a, or Let-7a mimic **(F)**.

We have previously shown that Let-7a regulates AMPKα2 levels (Simino et al., 2021). As expected, HF-O presented higher Let-7a and lower *Prkaa2* (the gene that encodes AMPKα2) hepatic transcript levels at baseline (Figures 3A,B). Also, AMPKα2 and pAMPKα2 protein levels were lower in the liver of HF-O compared to CT-O at baseline (Figure 3C). However, although Let-7a levels were upregulated in the liver of HF-O 48 h after PHX, AMPKα2 mRNA and protein levels did not differ between groups (Figures 3A–C). Let-7a is also predicted to bind to *Tgfβr1* and *Yap1* mRNA 3′UTR (Supplementary Table S3). YAP1 protein complexes with TAZ and acts as a transcription factor of genes related to cell proliferation (Nguyen-Lefebvre et al., 2021). YAP1 mRNA and protein levels did not differ between HF-O and CT-O at baseline. Nonetheless, 48 h after PHx, YAP1 gene expression and protein content were downregulated in the liver of HF-O (Figures 3D,E).

Flow cytometric analysis of AML12 cells showed that Let-7a levels may affect the hepatocytes’ ability to progress in the cell cycle, since Let-7a anti-miR led to higher cell number at the S-phase, similar to a negative control (15.4% *vs*. 13.3%, respectively), while Let-7a mimic led to fewer cells in the S-phase compared to a negative control (6.9% Let-7a mimic *vs*. 13.3% NC) (Figure 3F).

### Hepatocyte Proliferation and Liver Mass Recovery of Offspring Programmed by Maternal Obesity After Partial Hepatectomy

**FIGURE 4:**
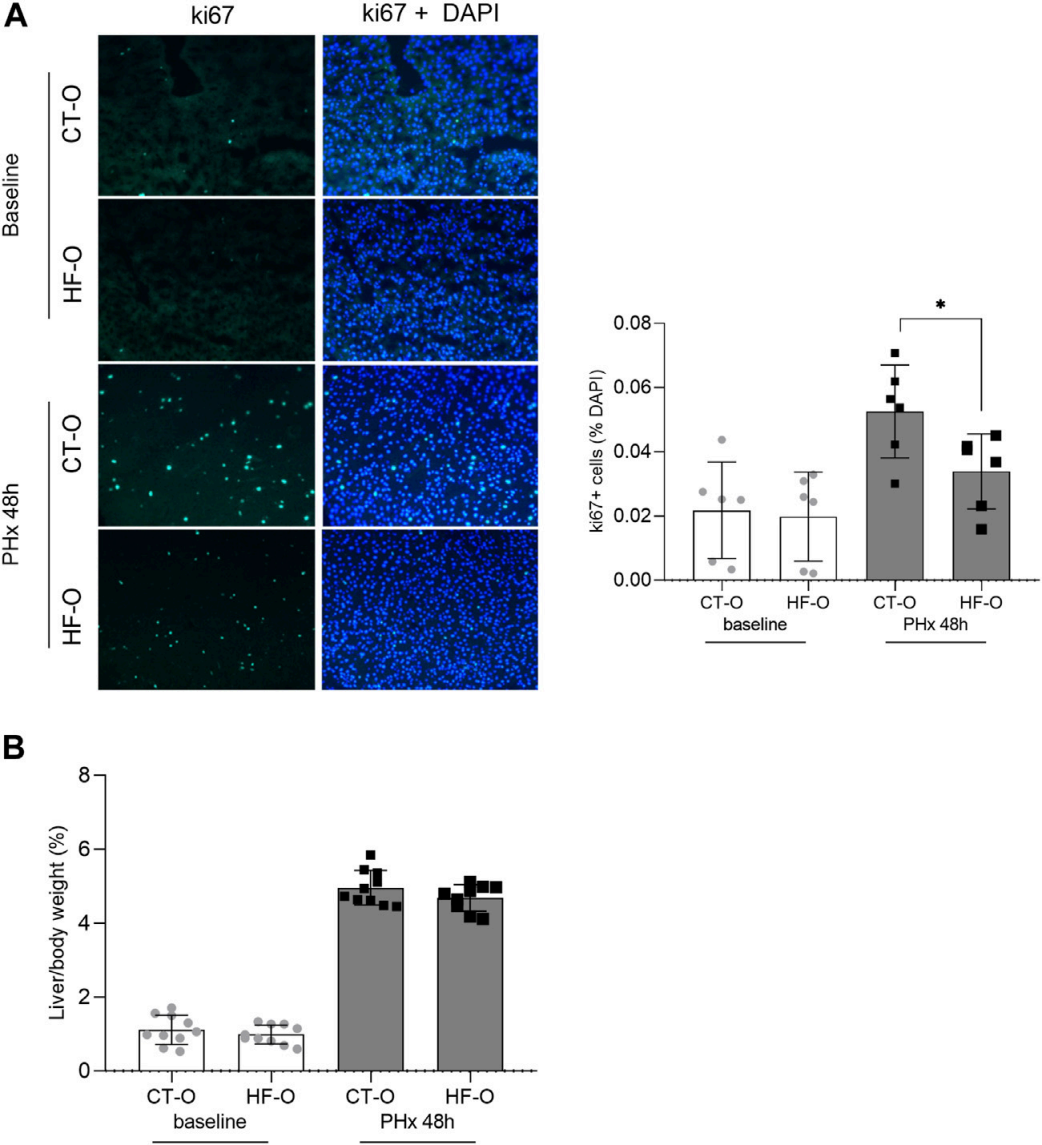
Liver regeneration capacity from the offspring of control and obese dams 48 h after partial hepatectomy. Immunofluorescence of hepatic ki67 content **(A)** and liver/body weight ratio **(B)** from the offspring of control (CT-O) and obese (HF-O) dams, 48 h (PHx 48 h) after partial hepatectomy surgery. Data are plotted as individual values (dot plots), means and SEM, represented by vertical bars. *n* = 6–10/group. *p ≤ 0.05 and **p ≤ 0.005 after Student’s *t*-test (CT-O versus HF-O).

To determine the liver regeneration capacity from the offspring of control and obese dams, we performed ki67 labeling of liver sections 48 h after PHx. HF-O had lower ki67 labeling 48 h after PHx, compared to CT-O (Figure 4A). However, the liver mass was similar between CT-O and HF-O 48 h after PHx because the liver/body weight ratio did not differ between the groups (Figure 4B).

## Discussion

This study sought to explore the relationship between miR-122, miR-370, and Let-7a expression with liver regeneration capacity in metabolically programmed offspring. We have confirmed that the development of an obese phenotype by HFD-fed dams leads the offspring to dysregulation of hepatic miR-122, miR-370, and Let-7a expression patterns and the predicted target genes involved with proliferation and regeneration. We have also shown altered dynamics of the cell cycle in hepatocytes transfected with mimic for Let-7a and miR-370, which reinforces the role of these miRNAs in the regenerative process. Although both the microRNAs and their targets were altered into the offspring of obese dams, most of them were comparable to CT-O after liver resection through ⅔ partial hepatectomy. In addition, despite the Ki67 labeling reflecting some delay in cell proliferation in HF-O, the liver mass recovery was not compromised, supporting the hypothesis of epigenetic reprogramming mechanism. To our knowledge, this is the first study that demonstrates the impact of obesogenic metabolic programming on epigenetic regulators that leads to the dysregulation in energy and lipid homeostasis and its role in liver regeneration.

There is much evidence that maternal obesity and/or high-caloric diet consumption during pregnancy and lactation can promote adverse metabolic outcomes in offspring (Zambrano et al., 2010; Alfaradhi et al., 2014; Benatti et al., 2014; Melo et al., 2014; Gaillard, 2015; Fante et al., 2016; Simino et al., 2017; Costa et al., 2020). Among the most well-characterized alterations in the liver of the offspring of obese dams, the development of NAFLD and its interconnection with insulin resistance has been widely explored (Watt et al., 2019; Tanase et al., 2020).

The liver is the main metabolic site and an important organ in immunity, and challenges in the perinatal period can lead to functional adaptations (Nakagaki et al., 2018). Maternal overnutrition during development can disturb the offspring’s hepatic function. In an elegant study developed by McCurdy et al. (2009), it was shown in non-human primates that maternal HFD consumption induced steatosis, oxidative stress, and apoptosis in liver fetuses, compromising liver function (McCurdy et al., 2009). The same was observed by Heerwagen et al. (2013) in mice at embryonic day 18.5 (Heerwagen et al., 2013). Additionally, premature weaning *per se* profoundly impacted the expression of several hepatic metabolic pathways leading to reprogramming in liver structure and function (Nakagaki et al., 2018).

The liver has a unique ability to regenerate (Mao et al., 2014), though the excessive presence of ectopic fat is a limiting factor for liver regeneration (Truant et al., 2013; Amini et al., 2016). Notably, our recent study showed that the offspring of obese dams have delayed regenerative process after partial hepatectomy; however, they seem to be programmed by maternal overnutrition to overlap the metabolic damage and promote cellular proliferation to ensure survival (Fante et al., 2021). However, this previous study did not assess the epigenetic modulation in the offspring of obese dams and the role of miRNAs in liver regeneration. The current study demonstrates that partial hepatectomy can induce changes in miRNA that impact lipid metabolism in liver offspring, reversing the expression of target genes involved in the regenerative process.

We have previously shown that maternal obesity induced by HFD consumption during gestation and/or lactation drives miRNA modulation in the liver of the offspring, which predisposes them to ectopic lipid accumulation and systemic metabolic alterations, consistent with NAFLD (Benatti et al., 2014; Simino et al., 2017; Simino et al., 2021). The offspring of obese dams showed upregulation of hepatic miR-370 and downregulation of miR-122 (Benatti et al., 2014). miR-122 is the most abundant miRNA in the liver that targets key genes involved in lipid and insulin homeostasis, thus playing an important role in general metabolic functions (Hsu et al., 2012; Tsai et al., 2012; Yang et al., 2012). miR-370 is also of great importance for liver metabolic homeostasis since it targets genes involved in fatty acids oxidation, such as *Cpt1a* and *Acadvl*, and controls miR-122 levels (Iliopoulos et al., 2010; Benatti et al., 2014; Simino et al., 2017). Similar results were obtained in a prenatal dexamethasone exposure (PDE) model. Liu et al. (2021) found increased lipid accumulation and molecular clues of NAFLD in the liver of adult male rat offspring consistent with low hepatic miR-122 expression.

Using bioinformatics approaches, we identified that *Nf2* (Neurofibromin 2) is a predicted target of miR-122. NF2 is known as a key component in the HIPPO signaling cascade, the major regulator of organ size/growth (Lee et al., 2021; Nguyen-Lefebvre et al., 2021). Upon acute tissue injuries, such as liver resection, NF2 no longer mediates YAP/TAZ (Yes-associated protein and transcriptional coactivator with PDZ-binding motif) complex phosphorylation and allows its recruitment to the nucleus, where it functions as a transcription factor for repair and regeneration processes (Lee et al., 2021; Nguyen-Lefebvre et al., 2021). At baseline conditions, HF-O had lower hepatic miR-122 levels, while *Nf2* gene expression was upregulated, which is an additional indicator that miR-122 can act as an *Nf2* repressor. It was already shown that transcripts associated with the Hippo signaling pathway, such as NF2, may be upregulated in obese mice and their descendants (An et al., 2017). However, here, after PHx, neither miR-122 nor *Nf2* levels were different in the liver of HF-O in comparison to CT-O. Although Nf2 gene expression dysregulation could be involved in NAFLD pathogenesis and progression to HCC (Ardestani et al., 2018), the reduction in the transcript levels in the HF-O group 48h after PHx, compared to baseline, may be an indication that the pathway that controls cell repair is more permissive in this group after mechanical injury, similar to the CT-O. This result is supported by the findings of cyclin D1 content in the same model in our previous work (Fante et al., 2021). Xiao et al. (2005) suggested that the product of the Nf2 gene inhibits cell cycle progression through Cyclin D1 suppression since Nf2 gene silencing results in upregulation of cyclin D1 and S-phase entry. We have shown that despite cyclin D1 showing a tendency to decrease in the HF-O group 48hs after PHx, the protein content still increases greatly in comparison to the baseline, being similar to the CT-O group and allowing the cell to enter the S-phase of the cycle, even with some delay (Fante et al., 2021).

The *Yap1* gene, of the YAP/TAZ complex, is reported here as a predicted target of Let-7a. We have previously shown that Let-7a is upregulated in the liver of the offspring of obese dams, and it targets *Prkaa2*, the gene that encodes AMPKα2 protein, leading to the disruption of the metabolic homeostasis (Simino et al., 2021). The Let-7 family is mainly known for its ability to regulate the expression of proteins involved in cell development, such as LIN28 (Reinhart et al., 2000) which has an important role in tissue repair (Shyh-Chang et al., 2013). The Let-7/Lin28 axis has been shown to be vital for the maintenance of the cell cycle in several conditions, and Let-7 overexpression may be detrimental for leading to the loss of cell growth and apoptosis induction (McDaniel et al., 2016). Let-7a levels were upregulated in the liver of HF-O at baseline and 48 h after PHx. However, attenuation on *Let-7* 4 h after PHx could confer the ability of the hepatic tissue to repair in circumstances of mechanical insult.

Lee et al. (2021) reported that several microRNAs may be able to suppress *Yap1* (miR-497, miR-186, miR-590-5p, miR-424-5p, miR-506, miR-132, mir_520c-3p, miR-375, miR-125b, miR-9-3p, miR-223, and miR-338-3p). On the other hand, Chaulk et al. (2014) suggested that YAP/TAZ could act as a transcription factor for Let-7 biogenesis since in MCF10A cells, Let-7 levels accumulated upon the loss of the nuclear YAP/TAZ. However, according to our research, this is the first study to suggest that Let-7a may pair with *Yap1*. *Yap1* gene expression and YAP/TAZ protein content did not differ between CT-O and HF-O at baseline, but 48 h after PHx, both *Yap1* expression and YAP/TAZ content were lower in the liver of HF-O, which would be consistent to *Let-7a* increasing.

The YAP/TAZ complex also interacts with other proteins and pathways involved in cellular proliferation and tissue regeneration. The dephosphorylation of YAP/TAZ and its consequent translocation to the nucleus allow it to form a complex with SMADS, which are key components of the TGFβ signaling pathway (Qin et al., 2018). In the current study, while *Tgfbr1* is shown to be a predicted target of Let-7a, *Tgfbr2* and *Smad3* are predicted targets of miR-370. TGFβ is known as an antiproliferative factor that plays an important role in the termination phase of the liver regeneration following partial hepatectomy since when the tissue has already been restored to its original size, hepatocyte growth must be inhibited to maintain constant liver mass and function (Qin et al., 2018; Nguyen-Lefebvre et al., 2021). On the other hand, high levels of TGFβ are associated with increased BMI (body mass index), metabolic damage, and higher serum glucose and insulin levels (Yadav et al., 2011; Tan et al., 2012). Although baseline TGFβ levels in HF-O were similar to the CT-O, 48 h after PHx, HF-O had higher TGFβ in both serum and the liver. Also, while the liver mass did not differ between groups, ki67 staining was slightly lower in the liver of HF-O 48 h after PHx, so we can correlate the upregulation of serum and liver TGFβ with a presumable marker of an efficient termination phase of the liver regeneration process.

*In vitro* experiments of gain and loss of function with antagomiR and mimic for Let-7a and miR-370 showed that these miRNAs are involved in the cell proliferation process since they were able to modulate different stages of the cell cycle. The transfection of hepatocytes with mimic for both Let-7a and miR-370 led to a reduction in the percentage of cells in the S-phase of the cell cycle. However, it is important to emphasize that even with some delay, the triggering of the regenerative process in the liver of offspring of obese dams still occurs as recently described by our laboratory (Fante et al., 2021).

In conclusion, repair mechanisms in metabolic programmed offspring occur through epigenetic reprogramming, thus ensuring tissue regeneration.

## Data Availability

The raw data supporting the conclusions of this article will be made available by the authors, without undue reservation.
