# Supplementary material for: Hepatic Epigenetic Reprogramming After Liver Resection in Offspring Alleviates the Effects of Maternal Obesity
Source: Front Cell Dev Biol. 2022 Mar 31;10:830009. doi: 10.3389/fcell.2022.830009 (PMC9009519; doi:10.3389/fcell.2022.830009)
Supplement: Supplementary file 1 [file DataSheet1.docx]

**Supplemental Table 1. Maternal Characterization**

|  | **CT** | | **HF** | |
| --- | --- | --- | --- | --- |
|  | Mean | SEM | Mean | SEM |
| Body weight (g) | 27.43 | 0.8023 | 35.09* | 1.472 |
| Adiposity (%) | 1.42 | 0.0936 | 2.35* | 0.0631 |
| Fasting glucose (mmol/L) | 7.10 | 0.1901 | 8.26* | 0.4105 |
| Serum insulin (pmol/L) | 110.8 | 15.82 | 245.2* | 28.54 |
| Serum CHOL (mmol/L) | 2.80 | 0.0615 | 4.22* | 0.1181 |
| Serum TAG (mmol/L) | 0.84 | 0.1014 | 1.13* | 0.0515 |

*  p ≤ 0.05

**Supplemental Table 2. d56 Offspring Characterization**

|  | **CT-O** | | **HF-O** | |
| --- | --- | --- | --- | --- |
|  | Mean | SEM | Mean | SEM |
| Body weight (g) | 36.8 | 0.6729 | 39.3* | 0.9564 |
| Adiposity (%) | 1.83 | 0.0937 | 2.51* | 0.2861 |
| Fasting glucose (mmol/L) | 4.97 | 0.4161 | 6.74* | 0.3722 |
| Serum insulin (pmol/L) | 93.35 | 4.044 | 112.4* | 8.054 |
| Serum CHOL (mmol/L) | 2.67 | 0.1065 | 3.02 | 0.3165 |
| Serum TAG (mmol/L) | 1.05 | 0.1008 | 2.04* | 0.1555 |

*  p ≤ 0.05

**Supplemental Table 3. ALT/AST at baseline, 4h and 48h post PHx**

**ALT AST**

|  | **CT-O** | | **HF-O** | | **CT-O** | | **HF-O** | |
| --- | --- | --- | --- | --- | --- | --- | --- | --- |
|  | **Mean** | SEM | **Mean** | SEM | **Mean** | SEM | **Mean** | SEM |
| **Baseline** | **25.32** | 2.960 | **25.32** | 3.087 | **20.08** | 1.285 | **27.50** | 1.764 |
| **PHx 4h** | **708.4** | 92.57 | **897.3** | 103.5 | **573.8** | 79.40 | **597.7** | 62.87 |
| **PHx 48h** | **58.93** | 21.42 | **40.74** | 7.345 | **88.83** | 11.17 | **65.26** | 3.676 |

**Supplemental Table 4. Pairing regions of microRNAs and predicted target mRNAs**

|  | Predicted consequential pairing - target region on top ( ) and miRNA on bottom ( ) |
| --- | --- |
| Position 715-722 NF2 3' UTR  mmu-miR-122-5p | 5' ...UACCAUGUGCCUCACACACUCCA...  \|\|\|\|\| \|\|\|\|\|\|\|\|  3' GUUUGUGGUAACAG----UGUGAGGU |
| Position 1587-1593 SMAD3 3' UTR  mmu-miR-370-3p | 5' ...AUGGUCCUCUGUUAUCAGCAGGG...  \|\|\|\|\|\|\|  3' UGGUCCAAGGUGGGGUCGUCCG |
| Position 61-68 TGFBR2 3' UTR  mmu-miR-370-3p | 5' ...GCCAAAGACCAGAGGCAGCAGGA...  \|\|\|\|\|\|\|  3' UGGUCCAAGGUGGGGUCGUCCG |
| Position 530-536 TGFBR2 3' UTR  mmu-miR-370-3p | 5' ...GCAACGAUCCCCUGACAGCAGGG...  \|\|\|\|\|\|\|  3' UGGUCCAAGGUGGG-GUCGUCCG |
| Position 2233-2239 of YAP1 3' UTR  mmu-let-7a-5p | 5' ...UGGUGCGCCUUGUUAUACCUCAA...  \|\|\|\|\|\|  3' UUGAUAUGUUGGAUGAUGGAGU |
| Position 66-73 of TGFBR1 3' UTR  mmu-let-7a-5p | 5' ...AGGAGGCUGGUUGUUCUACCUCA...  \|\|\|\|\|\|\|  3' UUGAUAUGUUGGAUGAUGGAGU |
| Position 3706-3712 of TGFBR1 3' UTR  mmu-let-7a-5p | 5' ...AGACCAAGGUACAUUUACCUCAU...  \|\|\|\|\|\|  3' UUGAUAUGUUGGAUGAUGGAGU |
